# Analyses of chlorogenic acids and related cinnamic acid derivatives from *Nicotiana tabacum* tissues with the aid of UPLC-QTOF-MS/MS based on the in-source collision-induced dissociation method

**DOI:** 10.1186/s13065-014-0066-z

**Published:** 2014-11-19

**Authors:** Efficient N Ncube, Msizi I Mhlongo, Lizelle A Piater, Paul A Steenkamp, Ian A Dubery, Ntakadzeni E Madala

**Affiliations:** Department of Biochemistry, University of Johannesburg, P.O. Box 524, Auckland Park, 2006 South Africa; CSIR Biosciences, Natural Products and Agroprocessing Group, Pretoria, 0001 South Africa

**Keywords:** *Nicotiana tabacum*, Cell suspensions, Chlorogenic acid, Cinnamic acid, ISCID, Leaf tissue, UPLC-qTOF-MS/MS

## Abstract

**Background:**

Chlorogenic acids (CGAs) are a class of phytochemicals that are formed as esters between different derivatives of cinnamic acid and quinic acid molecules. In plants, accumulation of these compounds has been linked to several physiological responses against various stress factors; however, biochemical synthesis differs from one plant to another. Although structurally simple, the analysis of CGA molecules with modern analytical platforms poses an analytical challenge. The objective of the study was to perform a comparison of the CGA profiles and related derivatives from differentiated tobacco leaf tissues and undifferentiated cell suspension cultures.

**Results:**

Using an UHPLC-Q-TOF-MS/MS fingerprinting method based on the in-source collision induced dissociation (ISCID) approach, a total of 19 different metabolites with a cinnamic acid core moiety were identified. These metabolites were either present in both leaf tissue and cell suspension samples or in only one of the two plant systems. Profile differences point to underlying biochemical similarities or differences thereof.

**Conclusion:**

Using this method, the *regio*- and geometric-isomer profiles of chlorogenic acids of the two tissue types of *Nicotiana tabacum* were achieved. The method was also shown to be applicable for the detection of other related molecules containing a cinnamic acid core.

## Background

Chlorogenic acids (CGAs) are the family of ester phytochemicals formed between cinnamic acid derivatives and quinic acids. These compounds are present in almost all plants and contribute a significant fraction of the total dietary intake of phenols in the daily human diet. Moreover, they possess some notable bio-medical or pharmacological properties [1-3]. CGAs are phenolic compounds produced through the shikimate- and phenylpropanoid pathways [4,5], and have been identified in responses against both biotic and abiotic stressors [6]. The most common naturally-occurring cinnamic acid derivatives that have been reported to be utilized during the biosynthesis of these molecules are *p-*coumaric -, caffeic- and ferulic acid which give rise to *p-*coumaroylquinic acid (*p*CoQA), caffeoylquinic acid (CQA) and feruloylquinic acid (FQA), respectively [1,2]. Other acids such as sinapic acids are, however, rarely found.

Naturally, plants are known to synthesize the *trans*-isomers over the *cis*-isomers of CGA compounds. The latter has been reported to be formed in tissue or extracts previously exposed to UV light, mechanical processing of coffee and electric field during MS data acquisition [7]. However, our latest study on tobacco cells treated with different chemical and biological priming inducers shows that the *cis*-isomer of 5-CQA was induced or up-regulated from a pre-existing pool when compared to non-treated cells. These results suggest the existence of possible enzymatic machinery responsible for the production of the *cis*-isomers in plants [8]. From an analytical perspective, CGA molecules offer a challenge owing to the structural similarities and complexity of these compounds. As such, scientists still spend a great deal of time on developing appropriate methodologies, even though reliable methods for the analysis have been developed in the past [8,9]. Most of the approaches for the analysis of these compounds are, however, achieved by the use of ion trap MS-based platforms [1,2,10-12]. Due to the limited availability of such instruments, emphasis is being placed on the development of alternative, equivalent methods to overcome these challenges. The development of the ion trap hierarchical approach has significantly contributed to the detailed analyses of CGA molecules [10-14]. Apart from the structural hierarchy, there exists *regio-* and geometric-isomerism which offers yet another dimension of complexity to the analyses of these molecules. However, the use of such ion trap-MS methods has also enabled the discrimination between *regio*- but not geometric-isomers of CGAs. As such, chromatographic methods have also been optimized for proper annotation. The order of elution of the *mono*-acylated CGA seems to remain constant on a reverse-phase column [12]. Using this knowledge, other methods based on Q-TOF-MS have been developed [7], albeit in most cases with several difficulties which ultimately affect the accuracy of the annotated metabolites.

To circumvent this problem, we have recently developed a Q-TOF-MS fingerprinting method based on the in-source collision induced dissociation (ISCID) approach for the analysis of chlorogenic acid derivatives [8]. This method has proved to generate very stable and reproducible results compared to previously published data. To substantiate our method, the current study profiles the CGA content of tobacco plant systems (leaf tissue vs. cultured cells) which have been reported to be a good source of a variety of bioactive constituents including CGAs [15].

For plant-related studies, mass production of secondary metabolites can be achieved by using plant cell suspensions. Cultured cells do not only provide a cost-effective alternative as they are also environmentally friendly. The main advantage of using this system is that it can be easily manipulated for biotechnological purposes [16,17]. However, it should be noted that cells in suspension culture differ significantly from mature organ tissue such as leaves in that cells grow rapidly and divide, do not contain any traces of chlorophyll and multiply in an aqueous environment containing stimulatory phytohormones [18]. Thus, the response to stress factors encountered by intact plant leaves and cell suspensions may vary. The evident biochemical differences between cell suspensions and leaf tissues have also been attributed to the different environmental conditions to which they are exposed to [19]. Furthermore, it has been reported that the biosynthesis of CGAs is highly dependent on the developmental stage of the tissues [20]. Cell type-associated localization of CGAs during maturation (*i.e.* tissue development) has also been reported [21].

As such, the aim of the current study was to comprehensively profile the CGA content of both tobacco leaf tissue and cell suspensions. The results of the current study are also expected to contribute to the identification of any underlying biochemical differences with regard to CGA biosynthesis between the two systems.

### Experimental

#### Plant material

*Nicotiana tabacum* cv. Samsun cell cultures were grown in a Murashige and Skoog (MS) medium containing 0.25 mg/L 2,4-dichlorophenoxyacetic acid and 0.25 mg/L kinetin (pH 5.8) [22] at room temperature on a shaker at 120 rpm with a light/dark cycle of 12 h/12 h, and low light intensity of 30 μmol/m^2^/s. Tobacco plants were grown in composted soil under greenhouse conditions: temperature min 10°C and max 22°C, light/dark cycle of 12 h/12 h, and light intensity of 60 μmol/m^2^/s.

#### Extraction of metabolites

The cells were harvested by filtration on 55 mm filter paper circles using a vacuum filtration system (Millipore, Billerica, MA, USA) and washed with 20 mL MS medium without vitamins while tobacco leaves were ground with a mortar and pestle in liquid nitrogen. Two grams (2 g) of each sample was weighed and homogenized in 20 mL (1:10 m/v) 80% methanol using a probe sonicator (Bandelin Sonopuls, Germany) set at 55% power for 15 sec with 4 cycles. The crude extract was centrifuged at 4100 x *g* for 15 min at room temperature. The supernatant was evaporated to approximately 1 mL using a rotary evaporator set at 55°C before being dried to completeness in a heating block set at 55°C overnight. The dried residues were reconstituted in 300 μL 50% (v/v) UHPLC-grade methanol in milli-Q water, and filtered through a 0.22 μm nylon filter into glass vials fitted with 500 μL inserts. The filtered extracts were stored at −20°C until analysis. For reproducibility of the results, the experiments consisted of 5 independent biological repeats and each extract was analyzed in duplicate (2 technical repeats).

#### Ultra-high performance liquid chromatography (UPLC)

The extracts were chromatographically analyzed on a UHPLC high-definition quadrupole time-of-flight MS instrument (UPLC-qTOF SYNAPT G1 HDMS system, Waters Corporation, Manchester, UK) fitted with an Acquity HSS T3 column (1.7 μm, 2.1 × 150 mm; Waters Corporation). A binary solvent system consisting of eluent A: 0.1% formic acid in water and B: 0.1% formic acid in acetonitrile (Romil Chemistry, UK) was used. A 20 min gradient method at constant flow rate of 0.4 mL/min was used for analyte separation, and the conditions were: 2% B over 0.0-1.0 min, 2-3% B over 1.0-3 min, 3-8% B over 3–4 min, 8-12% B over 4–12.00 min, 12 – 95% B over 12–13 min and held constant at 95% B over 13–15 min to wash the column and 95-5% B over 15–16 min. Thereafter, the column was returned to initial conditions at 16 min and allowed to equilibrate for 4 min. Chromatographic separation was monitored using a photodiode array (PDA) detector (Waters Corporation, UK) with a scanning range set between 200–500 nm, 1.2 nm bandwidth resolution and a sampling rate of 20 points/sec.

#### Quadrupole time-of-flight mass spectrometry (Q-TOF-MS)

Post-PDA detection, the metabolites were further detected with the aid of a SYNAPT G1 high definition mass spectrometer (Waters Corporation, UK) operating in negative ionization mode. The MS conditions were as follows: capillary voltage of 2.5 kV, sample cone voltage of 30 V, microchannel plate (MCP) detector voltage of 1600 V, source temperature of 120°C, desolvation temperature of 450°C, cone gas flow of 50 L/h, desolvation gas flow of 550 L/h, *m/z* range of 100–1000, scan time of 0.2 sec, interscan delay of 0.02 sec, mode set as centroid, lockmass set as leucine enkephalin (554.2615 Da), lockmass flow rate of 0.1 mL/min, and mass accuracy window of 0.5 Da. High purity nitrogen was used as desolvation gas, cone - and collision gas.

#### In-source collision-induced dissociation (ISCID)

Tandem MS (MS^2^) fragments of the chlorogenic acids were generated by an ISCID approach. For quinic acid bearing molecules, the approach was optimized for the detection of *m/z* 191 [quinic acid-H^+^] and a similar method was used for the other molecules herein. For CGA characterization the trap collision (30 eV) and cone energy (60 V) was experimentally optimised until stable fragmentation was obtained characterized by the formation of C1 [caffeic acid-H], C2 [caffeic acid-CO_2_], Q1 [quinic acid-H] and Q2 [quinic acid-H_2_O] ions (for CQA as an example).

## Results and discussion

**Figure 1:**
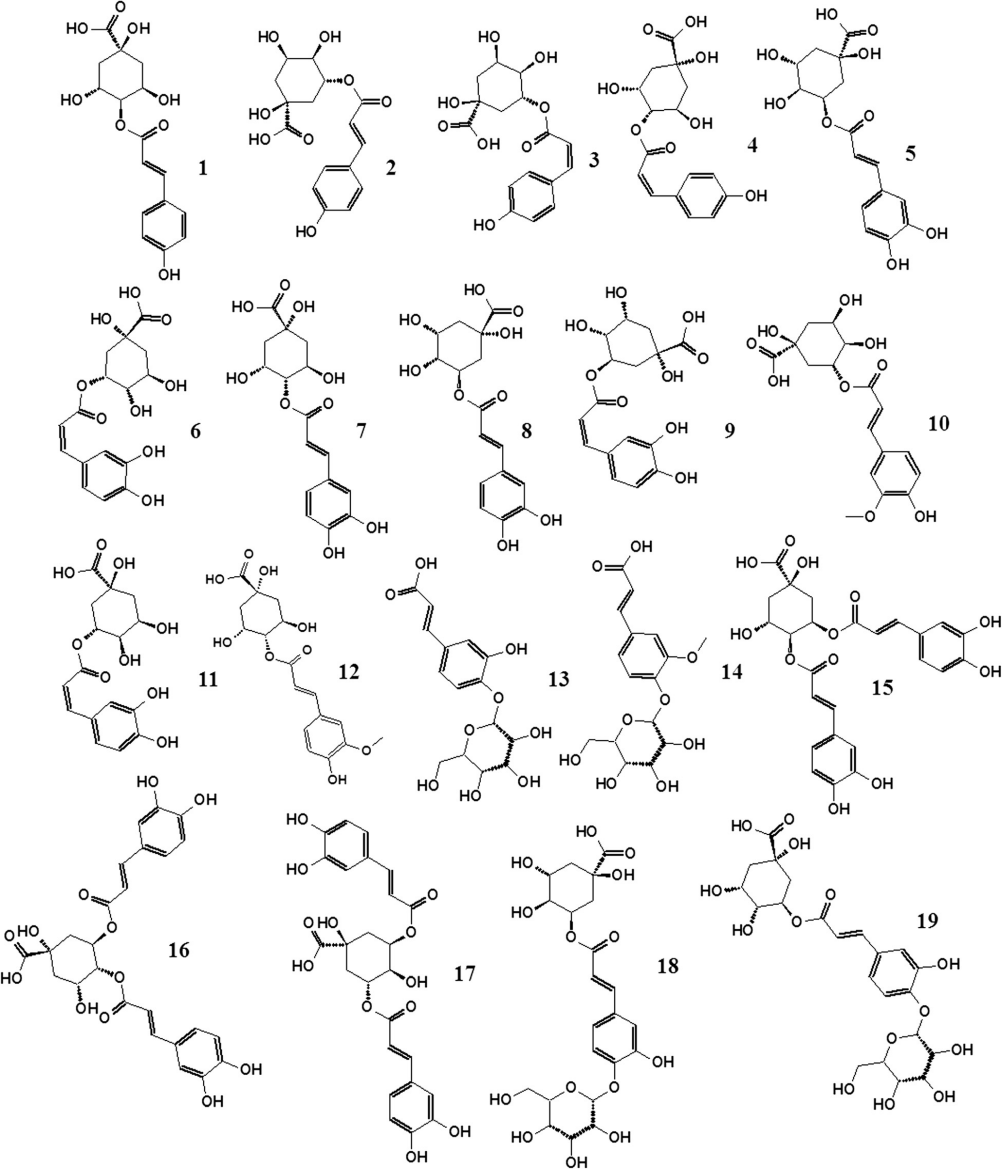
**The structures of chlorogenic acids and related derivatives and CQA glycosides detected in tobacco leaves and cultured cells.**

**Figure 2:**
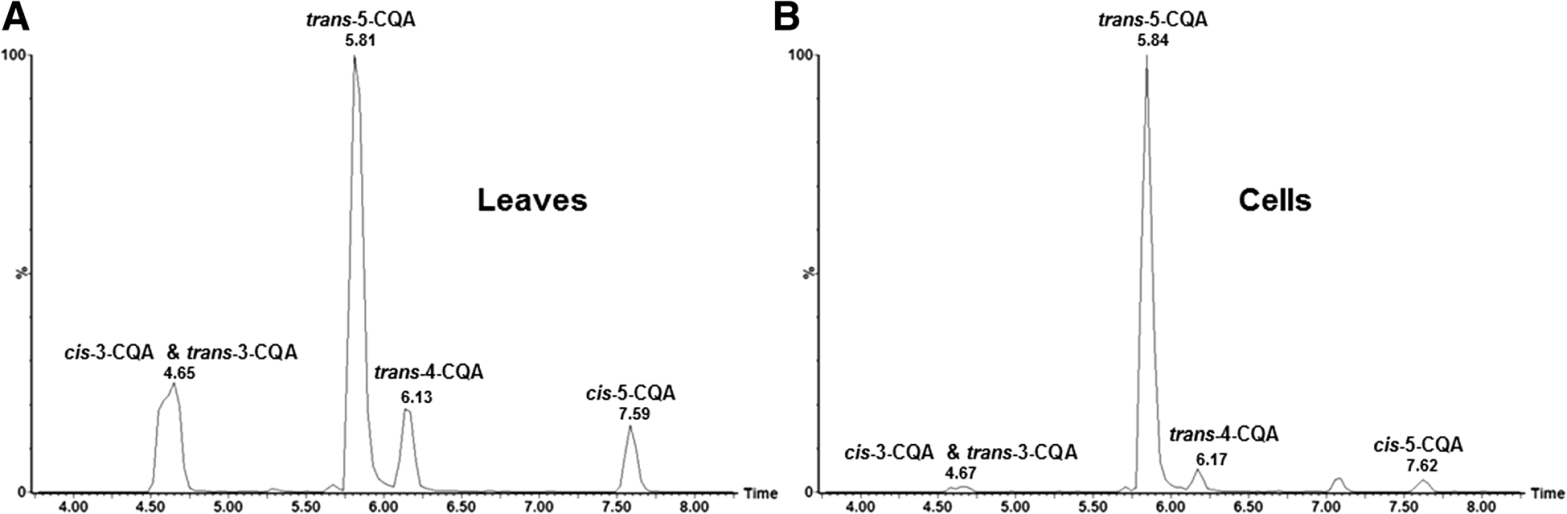
**Representative extracted single ion chromatograms (XIC) of UPLC-MS/MS showing differential elution times of** ***regio*** **- and geometrical isomers of CQAs in methanol extracts of tobacco leaf tissue (A) and cultured cells (B).** The *cis*-3-CQA and *trans*-3-CQA co-eluted at Rt =4.6-4.7 min.

**Figure 3:**
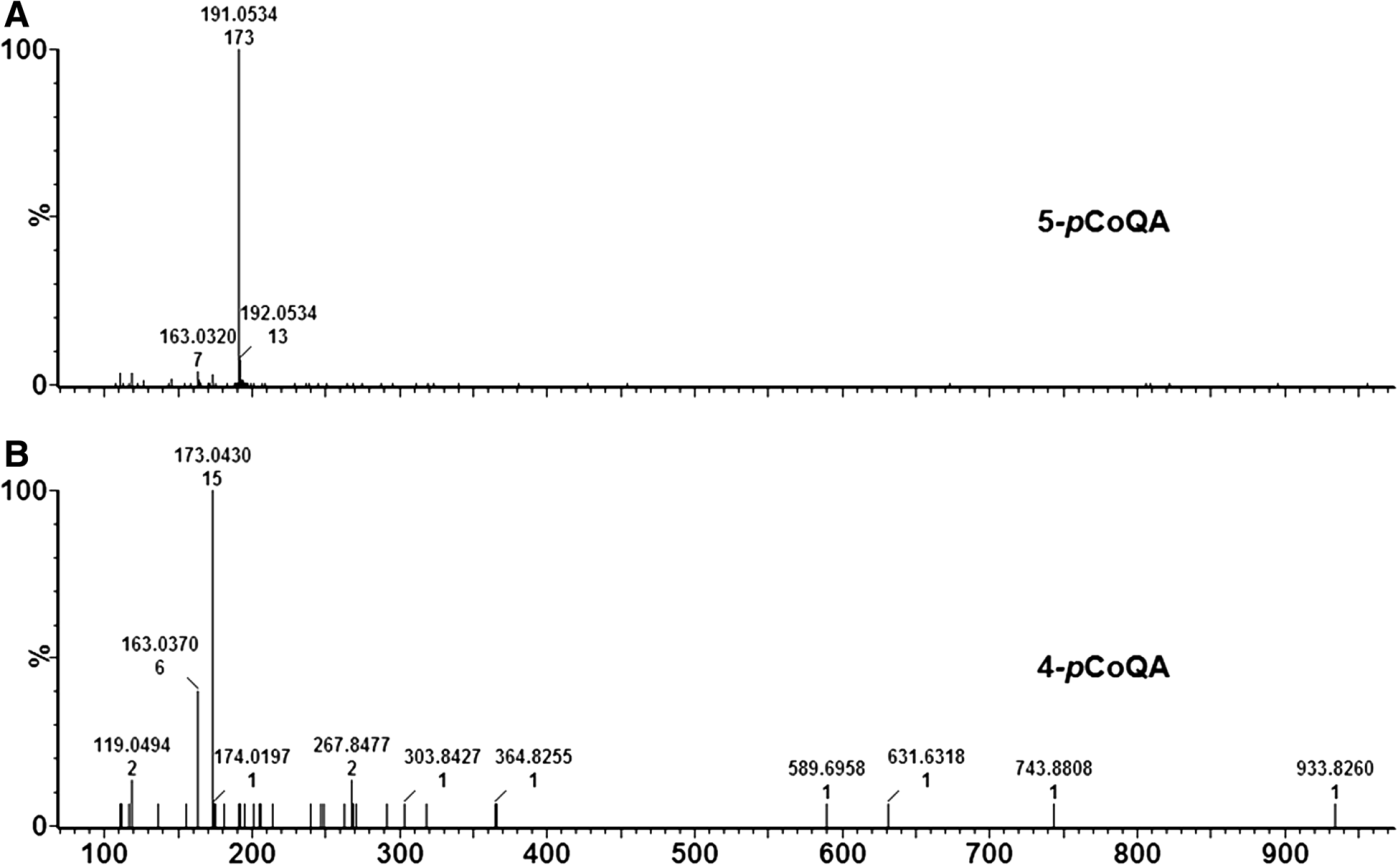
**Typical mass spectra of the fragmentation patterns of 5-** ***p*** **CoQA (A) and 4-** ***p*** **CoQA (B).**

**Figure 4:**
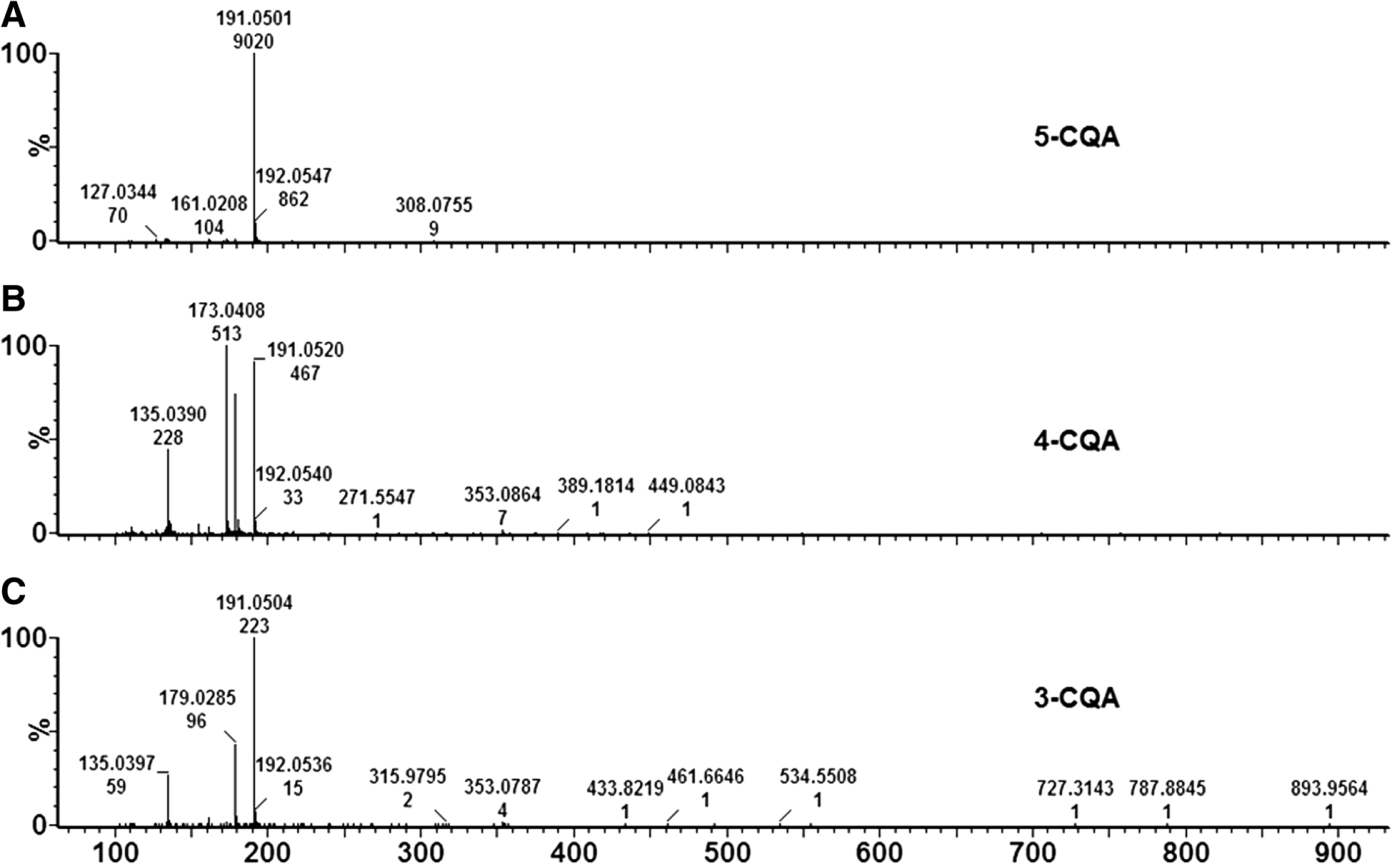
**Typical mass spectra of the fragmentation patterns of 5-CQA (A), 4-CQA (B) and 3-CQA (C).**

**Figure 5:**
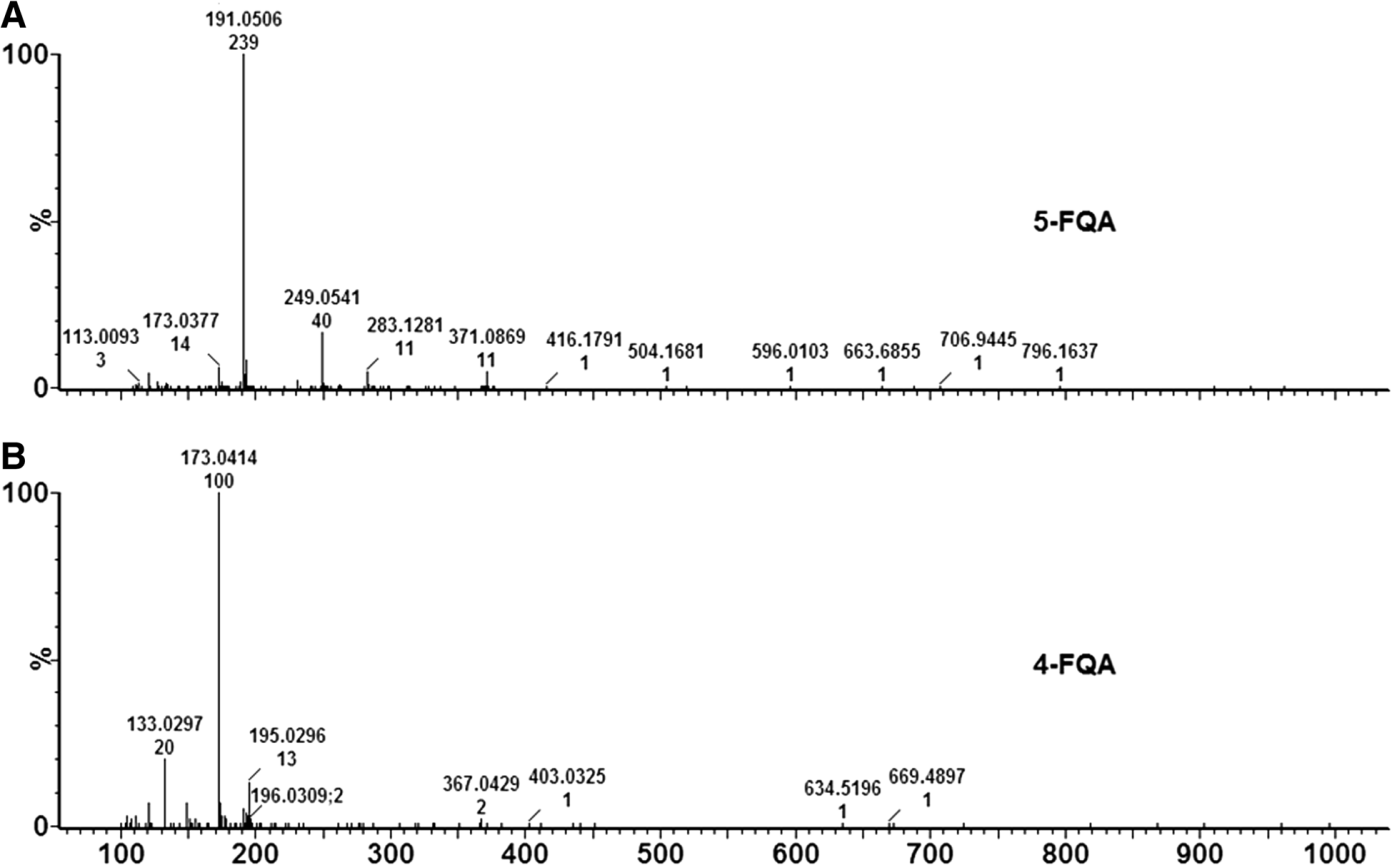
**Mass spectra of the fragmentation patterns of 5-FQA (A) and 4-FQA (B).**

**Figure 6:**
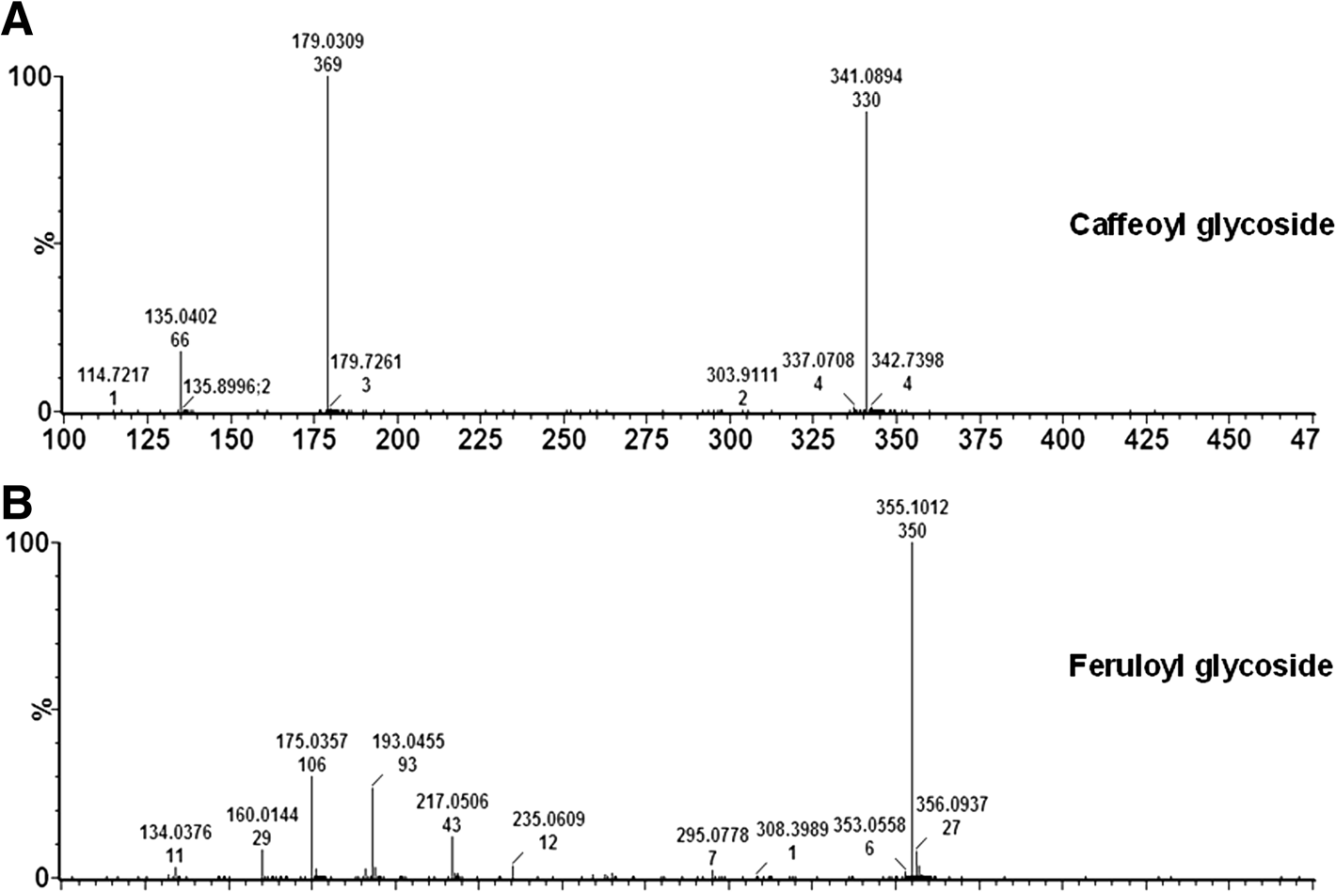
**Mass spectra of the fragmentation patterns of caffeoylglycoside (A) and feruloylglycoside (B).**

**Figure 7:**
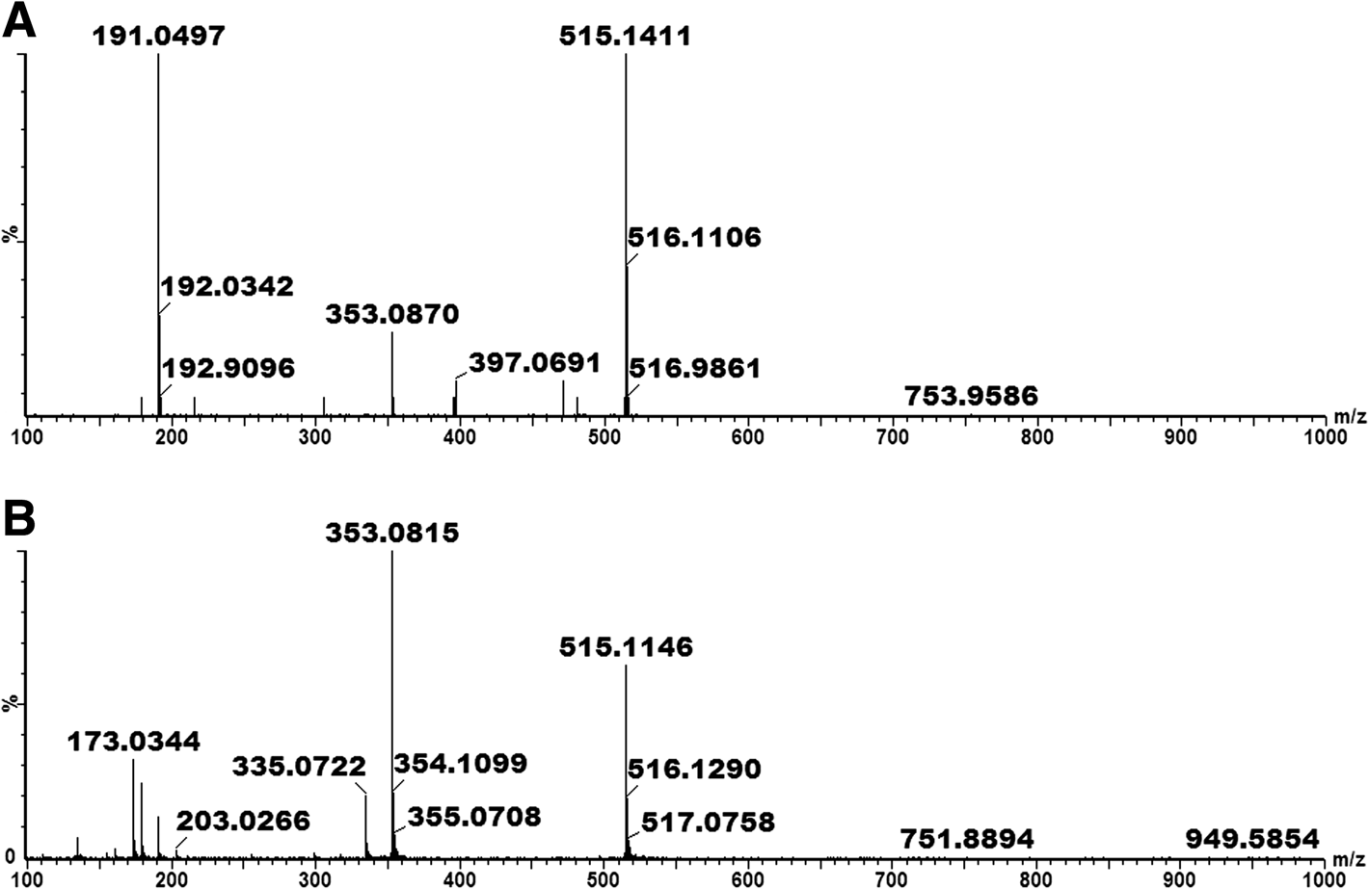
**Mass spectra of the fragmentation patterns of 3,5-** ***di*** **-CQA (A), 4,5-** ***di*** **-CQA (B).**

Due to the lack of authentic standards and low concentration of metabolites, the current study only presents qualitative data. In most metabolite fingerprinting studies, metabolite identification is challenging and requires the use of authentic standards for definite metabolite identification. However, due to unavailability of most plants standards, we opted for cider and coffee as surrogate extracts for some of the metabolites identified herein [12]. It is also worth mentioning that the introduction of instruments such as Q-TOF-MS have contributed significantly in overcoming this problem because of its ability of measuring mass accuracy below 3 ppm [23]. For all the 19 metabolites detected (Figure 1, Table 1), the mass error was below 5 ppm. As previously mentioned, proper annotation of structurally similar isomers of cinnamic acid derivatives is a difficult task [9]. To avoid ambiguity during identification, using the IUPAC numbering system, we have considered all possible factors ranging from chromatographic separation to mass spectrometric behaviour as shown in previously published data [10,12]. In order to simplify identification, metabolites harboring common cinnamic acid moieties in their core structure were monitored using a single mass extracted ion chromatogram (XIC). As an example, four peaks with *m/z* 353, representative of three *regio*-isomers of CQA, were detected in extracts from both cells and leaves (Figure 2). This enabled proper identification of CGAs based on previously published data, taking into consideration all possible isomers both at positional and geometrical level [1,2,10-12,23]. It has been reported that CGA molecules fragment in a similar manner under well-optimized conditions. For instance, CQAs are known to result in the following fragment ions: Q1 [quinic acid-H], C1 [caffeic acid-H], Q2 [quinic acid-H_2_O] and C2 [caffeic acid-CO_2_] [24]. By monitoring these ions, CGA and the related derivatives could be positively annotated as discussed below. All mass spectra shown in Figures 3, 4, 5, 6 and 7 were generated at a collision energy of 30 eV and 60 eV cone voltage as described under Experimental section.

**Table 1:** **Characterization of chlorogenic acids and related cinnamic acid derivatives detected in tobacco leaves and cells**

**Compound name**	**Retention time (min)**	**Cells**	**Leaves**	**[M-H]** ^**−**^	**Diagnostic** ***m/z*** **ions**
*cis*-4-*p*CoQA **(4)**	7.35	✓	✓	337.0933	173
*trans*-4- *p*CoQA **(1)**	7.96	✓	✓	337.0957	173
*trans*-5-*p*CoQA **(2)**	7.69	✓	✓	337.0886	191
*cis*-5-*p*CoQA **(3)**	10.54		✓	337.0949	191
*cis*-3-CQA **(6)**	4.55	✓	✓	353.0875	191, 179, 135
*trans*-3-CQA **(5)**	4.61	✓	✓	353.0787	191, 179, 135
*trans-*4-CQA **(7)**	6.13	✓	✓	353.0864	191, 179, 173, 135
*trans*-5-CQA **(8)**	5.81	✓	✓	353.0829	191, 135
*cis*-5-CQA **(9)**	7.55	✓	✓	353.0809	191, 135
*trans*-4-FQA **(12)**	6.17		✓	367.0429	173
*trans*-5-FQA **(10)**	5.84	✓	✓	367.0708	191
*cis*-5-FQA **(11)**	9.41	✓	✓	367.0847	191
Caffeoylglycoside **(13)**	5.60	✓	✓	341.0894	179
Feruloylglycoside **(14)**	7.09	✓		355.1012	193
3,4-*di*CQA **(15)**	5.21		✓	515.1509	191, 179, 173, 135
4,5-*di*CQA **(16)**	5.50	✓	✓	515.1404	191, 179, 135
3,5-*di*CQA **(17)**	6.30	✓	✓	515.1477	191, 179, 135
3-CQA glycoside **(18)**	4.78		✓	515.1261	191, 179, 135
5-CQA glycoside **(19)**	5.47		✓	515.1354	191, 135

### Characterization of *p*-coumaroylquinic acids

*p*-Coumaroylquinic acid has a molecular weight (Mr) of 338 and four peaks at *m/z* 337 were detected in leaf extracts whilst only three were detected in cells (XIC not shown). These ion peaks, according to the mass spectra (Figure 3), were identified as *trans*-4-*p*CoQA (**1**), *trans*-5-*p*CoQA (**2**), *cis*-5-*p*CoQA (**3**) and *cis*-4-*p*CoQA (**4**). The leaf samples were found to contain **1**, **2**, **3** and **4** while the cells were found to only contain **1**, **2** and **4**. The absence of **3** (*cis*-5-*p*CoQA) in cells could not be entirely explained but is possibly due to the fact that some molecules are more prone to isomerization than others, a phenomenon which has been observed in other plants but without supporting explanation [8]. In addition, another important factor to note is that both *trans-* and *cis-*isomers have very similar fragmentation patterns [12]. In the current study the mass spectra of the *trans* isomers are shown throughout. The notable absence of 3-*p*CoQA in both leaf and cell extracts is an observation which represents a very interesting biochemical phenotype. This molecule has been found to accumulate in other plants such as legume forages and birch trees [25-27].

### Characterization of caffeoylquinic acids

Although present at different relative intensities, all CQA *regio*-isomers (Mr = 354) were identified in both tobacco leaves and cultured cells: *trans*- and *cis*-3CQA (**5**, **6**), *trans*-4-CQA (**7**) and *trans*- and *cis*-5-CQA (**8**, **9**) (Figure 4). Amongst the CGAs, the CQAs have the widest occurrence and are well researched [21,28]. Unlike *p*CoQA, only 3- and 5-acylated CQA possessed both *cis*- and *trans*-isomers whilst only the *trans*-isomer of 4-CQA (**7**) was detected. All the mentioned CQA molecules were found in both leaf and cell suspension samples. Another interesting observation was the fact that *cis*-5-CQA (**9**) appeared in a relatively high intensity compared to the *cis*-3- CQA in both the leaf and cell samples. The same was also noted in our previous study where the same molecule was shown to be induced by activators of plant defence and priming responses [8]. The differences in the metabolite profiles (including CGAs and phenolic content) between cells and leaf tissue have been previously reported [21,29]. However, according to the best of our knowledge, this is the first report focusing on the differences between cells and leaf tissue samples taking into account both regional and geometrical isomerism (*cis* and *trans* configurations) of CGAs. From our previous work [8], we could confidently conclude that the *cis*-5-CQA molecule could be a natural product of tobacco plant systems, suggesting an interesting biochemical phenotype which is not fully explained in other plant species.

### Characterization of feruloylquinic acids

Feruloylquinic acids have an Mr = 368. Similarly to *p*CoQA and CQA, molecules harboring ferulic acid moieties were also identified (Figure 5). However, only three peaks were successfully identified; as *trans*-5-FQA (**10**), *cis*-5-FQA (**11**) and *trans*-4-FQA (**12**) respectively (Table 1). Similarly to the *p*CoQA, there was an absence of the 3-acyl molecule in both cell- and leaf extracts. These three FQAs were identified in leaf tissue, however, only *trans*-5-FQA (**10**) and *cis*-5-FQA (**11**) were identified in cells, suggesting an underlying biochemical difference between the two systems, possibly due to enzymes differing in their substrate specificities.

### Characterization of caffeoylglycoside and feruloylglycoside

Caffeoylglycoside and feruloylglycoside have molecular weights of 342 and 356 respectively. Two molecules with pseudomolecular peaks at *m/z* 341 **(**Figure 6A**)** were identified as isomers of caffeoylglycoside (**13**) in both leaf and cell samples. They produced distinctive ions at *m/z* 179 ([caffeic acid − H]−) by the loss of a glucosyl residue (C_6_H_10_O_5_) and *m/z* 135 ([caffeic acid − H] −). As for the feruloylglycoside at *m/z* 355 (**14**, Figure 6B), it was also tentatively identified based on its fragmentation patterns: briefly this molecule produced a base peak at *m/z* 193 ([ferulic acid − H]−) and by the loss of a glucosyl residue (C_6_H_10_O_5_) (162 Da). It also produced a base peak at *m/z* 175 ([ferulic acid – H – H_2_O]− ), a peak at *m/z* 295 ([M − H −60 Da]−) by the loss of C_2_H_4_O_2_ and another peak at *m/z* 235 ([M − H −120 Da]−) by the loss of C_4_H_8_O_4_, due to internal sugar fragmentations. The identification of these molecules was also found to be consistent with published data [9]. Interestingly, this molecule was only identified in cells and not in leaf samples. The fact that feruloylglycoside molecules are only biosynthesized in cell suspensions again suggests a very interesting biochemical characteristic of the cultured cells in suspension which is absent in the leaf tissue.

### Characterization of *di*-caffeoylquinic acid and caffeoylquinic acid glucoside

**Figure 8:**
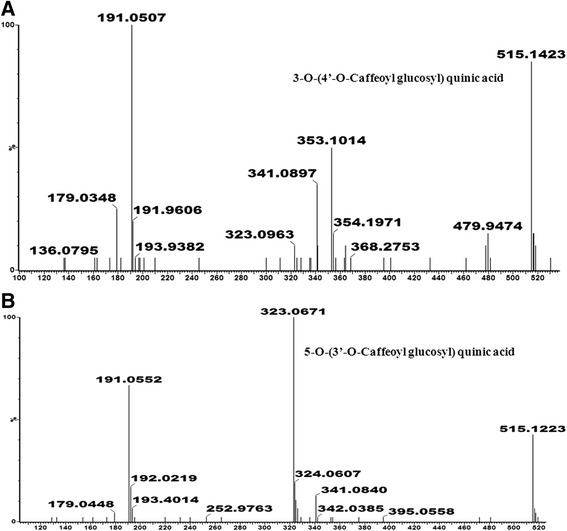
**Mass spectra of the fragmentation patterns of 3-** ***O-*** **(4’-** ***O-*** **caffeoyl glucosyl) quinic acid (A) and 5-** ***O-*** **(3’-** ***O-*** **caffeoyl glucosyl) quinic acid (B).**

Both *di*-caffeoylquinic acid and caffeoylquinic acid glucoside have an Mr =516. Here, a maximum of nine peaks at *m/z* 515 were identified and, based on the accurate masses and fragmentation patterns, these ions were distinguished as either *di*-caffeoylquinic acid and caffeoylquinic acid glycosides (Figure 7). As previously reported, both *di*-CQA and CQA-glycoside produce an isobaric pseudomolecular ion at *m/z* 515. As such, molecules **15**–**19** were annotated as either *di*-CQA or CQA glycosides. Based on the accurate mass, the *di*CQA were detected with an average *m/z* of 515.1463 (C_25_H_23_O_12_) and the CQA glycosides were found to have an average *m/z* of 515.1292 (C_22_H_27_O_14_). Based on information published elsewhere [10,11], molecules **15**–**17** were identified as *di*-CQAs (3,4 *di*-CQA (**15**), 4,5 *di*-CQA (**16**) and 3,5 di-CQA (**17**). Interestingly, both leaves and cells were found to contain these compounds except for the 3,4 *di*-CQA which was only present in the cells. Unlike the *di*CQA, the CQA glycosides produce distinctive ions at *m/z* 341 ([caffeoyl glucoside – H]−) or/and 323 ([caffeoyl glucoside – H – H_2_O]−) which were not present in the *di*CQA MS spectra. Recently, Jaiswal et al. [30,31] reported the hierarchical fragmentation scheme of similar molecules, briefly it was noted that CQA forms a glycoside through an ether bond at either C-3 or C-4 on the aromatic caffeoyl ring. During MS fragmentation, these molecules gives rise to ions at *m/z* 341 which predominates in both cases; however a peak at *m/z* 323 is a characteristic of glucosyl attachment at C-3 [30]. Thus these molecules were putatively identified as 3-*O-*(4’-*O-*caffeoyl glucosyl) quinic acid (**18**) since it also produced similar fragmentation to 3CQA. In turn, 5-*O-*(3’-*O-*caffeoyl glucosyl) quinic acid (**19**) produces fragmentation similarly to 5-CQA (Figure 8). Interestingly, the CQA glycosides were only present in leaf tissue samples. Also, a previous report showed the accumulation of similar glycosides in *Moringa* leaves [32] and recently in *Lonicera* leaves [30].

## Conclusion

Using the described UHPLC-Q-TOF-MS/MS fingerprinting method, based on the ISCID approach, a total of 19 different metabolites with a cinnamic acid core moiety were identified. Chlorogenic acids and related compounds are important because their involvement during plant defence responses is also becoming apparent [8,33,34]. In a separate study, the distribution of these molecules were found to be different across the different parts of the plants [35], which is an indication that there could be a localised function associated with a particular content and distribution. Similarly, the results of the current study indicate that there exists a significant difference in the CGA profiles of tobacco leaf tissue and cell suspensions. Possibly, the differences stem from different biochemical pathways leading to the biosynthesis of CGA molecules in the two biological systems. As already mentioned, the biochemical differences between cells and leaf tissue could be a result of the different environmental conditions which the cells and the leaves are exposed to as well as the level of tissue differentiation. This finding is in agreement with other published data where it was demonstrated that the CGA content varies with plant developmental stages. Therefore, there is a need to further investigate the underlying biochemical differences in such plant systems by investigating the same plant systems at transcriptomic and proteomic levels in conjunction with MS-based metabolite profiling.

## Abbreviations

CGA: Chlorogenic acids
CQA: Caffeoylquinic acid
UHPLC: Ultra-high performance liquid chromatography
ISCID: In-source collision-induced dissociation
Q-TOF-MS: Quadrupole time-of-flight mass spectrometry
